# Comatose Patients After Cardiopulmonary Resuscitation: An Analysis Based on Quantitative Methods of EEG Reactivity

**DOI:** 10.3389/fneur.2022.877406

**Published:** 2022-06-03

**Authors:** Huijin Huang, Yingying Su, Zikang Niu, Gang Liu, Xiaoli Li, Mengdi Jiang

**Affiliations:** ^1^Department of Neurology, Xuanwu Hospital, Capital Medical University, Beijing, China; ^2^State Key Laboratory of Cognitive Neuroscience and Learning & IDG/McGovern, Beijing Normal University, Beijing, China

**Keywords:** coma, EEG reactivity, quantification, entropy, connectivity

## Abstract

**Objective:**

Every year, approximately 50–110/1,00,000 people worldwide suffer from cardiac arrest, followed by hypoxic-ischemic encephalopathy after cardiopulmonary resuscitation (CPR), and approximately 40–66% of patients do not recover. The purpose of this study was to identify the brain network parameters and key brain regions associated with awakening by comparing the reactivity characteristics of the brain networks between the awakening and unawakening groups of CPR patients after coma, thereby providing a basis for further awakening interventions.

**Method:**

This study involved a prospective cohort study. Using a 64-electrode electroencephalography (EEG) wireless 64A system, EEG signals were recorded from 16 comatose patients after CPR in the acute phase (<1 month) from 2019 to 2020. MATLAB (2017b) was used to quantitatively analyze the reactivity (power spectrum and entropy) and brain network characteristics (coherence and phase lag index) after pain stimulation. The patients were divided into an awakening group and an unawakening group based on their ability to execute commands or engage in repeated and continuous purposeful behavior after 3 months. The above parameters were compared to determine whether there were differences between the two groups.

**Results:**

(1) Power spectrum: the awakening group had higher gamma, beta and alpha spectral power after pain stimulation in the frontal and parietal lobes, and lower delta and theta spectral power in the bilateral temporal and occipital lobes than the unawakening group. (2) Entropy: after pain stimulation, the awakening group had higher entropy in the frontal and parietal lobes and lower entropy in the temporal occipital lobes than the unawakening group. (3) Connectivity: after pain stimulation, the awakening group had stronger gamma and beta connectivity in nearly the whole brain, but weaker theta and delta connectivity in some brain regions (e.g., the frontal-occipital lobe and parietal-occipital lobe) than the unawakening group.

**Conclusion:**

After CPR, comatose patients were more likely to awaken if there was a higher stimulation of fast-frequency band spectral power, higher entropy, stronger whole-brain connectivity and better retention of frontal-parietal lobe function after pain stimulation.

## Introduction

Hypoxic-ischemic encephalopathy (HIE) after cardiopulmonary resuscitation (CPR) is a major health problem. Approximately 50–110 people/100,000 people worldwide have cardiac arrest every year (1). Although some patients recover consciousness, 40–66% of them fail to awaken, resulting in a long hospitalization period and high economic burden, which makes early prediction of awakening the focus of neurocritical care research and plays a vital role in medical decision-making (2, 3). Evoked potentials (EPs), which are a reliable way of measuring the functional status of the brain, have been used to predict the prognosis of patients who are comatose for more than 50 years. Some studies have found that the bilateral absence of the N20 component was the most discriminating predictor with a specificity of 100% for an unfavorable outcome (4, 5). The combination of the N60 and mismatch negativity (MMN) offered good predictive performance for awakening with increased sensitivity (70%) and improved specificity (91.7%) (6). However, the EPs are confounded by cervical spinal cord injury and isolated brain stem lesions. Also, interpretation of EPs signal are prone to artifacts interference. As a simple and safe bedside monitoring and analysis technology, electroencephalography (EEG) has been widely used in coma research. Slow-wave EEG patterns and the presence of EEG reactivity (EEG-R) can predict a good prognosis with a sensitivity of 42~97% and specificity of 92~100% (7, 8). EEG-R is defined as any reproducible change in the frequency or amplitude of the EEG background pattern after external stimulation (9). External stimulation can consist of auditory (clapping, loud name calling), somatosensory or nociceptive (pinching limbs or nipples, squeezing nails or bone periosteal surface), or visual (spontaneous or forced eye opening, intermittent light stimulation) stimulation (9, 10). In 2018, a systematic review found that regardless of the etiology, patients with impaired consciousness featuring a reactive EEG were more likely to have a favorable outcome. EEG-R is a valuable prognostic parameter and warrants rigorous assessment (11).

However, EEG-R is not only characterized by its presence or absence. In addition to reflecting the integrity of the conduction pathway, EEG-R is also the result of the brain's processing of various stimuli. Therefore, there are also differences in the direction, degree and internal relationship of changes, and the visual analysis is too subjective. Thus, researchers have started to focus on EEG-R quantitative and network analyses. Previous studies have found that quantitative analysis (power spectrum) of EEG-R is better than visual analysis and can well predict recovery after coma (sensitivity 90%, specificity 88%) (12–14). In 2020, our team used 16-electrode EEG to analyze the brain network characteristics of EEG-R in comatose patients. It was found that patients who regained consciousness had higher alpha coherence in the posterior cortex and lower delta coherence in the central brain cortex than patients with a poor prognosis (15). This study provided reliable parameters for coma prognosis; however, due to the small number of channels, the construction of the network was not accurate enough, and it is impossible to more precisely identify the brain areas related to awakening. Therefore, the purpose of this study was to identify the parameters and key brain regions associated with awakening and to provide a basis for the development of awakening interventions by comparing the differences in the quantitative analysis and EEG-R brain network characteristics between an awakening group and an unawakening group of CPR patients after coma.

## Materials and Methods

### Materials

Between 2019 and 2020, comatose patients were enrolled in the neurocritical care unit (NCCU) at Xuanwu Hospital of Capital Medical University. The inclusion criteria were as follows: (1) patients were 18–80 years of age; (2) initial assessments were conducted within 1 month of symptom onset; (3) for patients who received targeted temperature management, the clinical and EEG assessments were performed during normothermia after therapeutic hypothermia and sedation; and (4) the Glasgow coma scale (GCS) score was ≤ 8. The exclusion criteria were as follows: (1) premorbid conditions such as neurological or psychological diseases, brain trauma or surgery; (2) within 5 half-lives for all CNS-depressing medications (such as anesthetics, sedatives and antipsychotics) before data collection; (3) simultaneous multiple organ dysfunction with unstable vital signs; (4) peripheral neuropathy or spinal cord lesions affecting afflux of pain stimulus [detected by medical history and examinations such as electromyography (EMG), somatosensory evoked potentials (SSEP) and magnetic resonance imaging (MRI)] and (5) a failure to follow up. The study observed the principles of the Helsinki Declaration and was approved by the Ethics Committee of Xuanwu Hospital, Capital Medical University, Beijing. Informed consent was obtained from the family members or designated surrogates of all participants.

### Methods

This trial was designed as a prospective, blinded cohort study and was based on our previous study, which found parameters that were closely related to consciousness (16). The participants were divided into the awakening group or the unawakening group based on whether they recovered consciousness; the patients were assessed by two experienced neurologists who were blinded to the EEG assessments. The awakening of patients was defined as the patient's ability to clearly show discernible evidence of self or surrounding awareness by demonstrating the ability to carry out commands or exhibit reproducible and sustained purposeful behavior (17). The prognosis was assessed using the Cerebral Performance Category (CPC) (Table 1), and the patients were divided into the awakening group (CPC 1–3) or the unawakening group (CPC 4–5) (18).

**Table 1:** Cerebral performance category (CPC) score scale.

**CPC 1**	**Good cerebral performance: conscious, alert, able to work, might have mild neurologic or psychologic deficit**.
CPC 2	Moderate cerebral disability: conscious, sufficient cerebral function for independent activities of daily life. Able to work in sheltered environment.
CPC 3	Severe cerebral disability: conscious, dependent on others for daily support because of impaired brain function. Ranges from ambulatory state to severe dementia or paralysis.
CPC 4	Coma or vegetative state: any degree of coma without the presence of brain death criteria. Unawareness, even if appears awake (vegetative state) without interaction with environment; may have spontaneous eye opening and sleep/awake cycles. Cerebral unresponsiveness.
CPC 5	Brain death: apnea, areflexia, EEG silence, etc.

### Clinical Evaluation

A clinical evaluation was performed by at least 2 NCCU professional physicians to characterize the included participants. The NCCU physicians who recorded the general information of the participants, including age, sex, duration of arrest or resuscitation, time from coma after cardiac arrest to EEG assessment, GCS and contact information, were blinded to the EEG results. The researchers who analyzed the EEG results were blinded to the clinical conditions.

### EEG Assessment

#### EEG Recording

EEG data were recorded at the bedside within 1 month of illness onset with NicoletOne software (Nicolet, America) using a 64-electrode EEG wireless 64A system. For each patient, EEG was recorded at least once and lasted for 30 to 60 min. Electrodes were placed according to the international 10–10 system. The data were referenced to the Cpz electrode. Impedances were maintained at <5 kΩ. The continuous EEG data were recorded online at a sampling rate of 512 Hz with a bandpass filter in the range of 0.5–70 Hz and a 50 Hz notch filter. The EEG reactivity test used painful stimulation (nail bed pressure), and a simultaneous stimulation mark was performed on the EEG segment. The stimulation lasted 10 s, and the observation time was at least 30 s. During the EEG acquisition period, all instruments and equipment that might interfere with EEG signals were switched off to ensure that the surroundings were quiet and stable and to avoid signal fluctuations and artifact interference.

#### Pre-processing

First, a 0.5~45 Hz bandpass digital filter was used to attenuate frequency artifacts. Noise caused by eye movement was removed by using the FastICA algorithm. Then, the superfast spherical interpolation method was used to interpolate the bad channels. After that, all of the signals were converted to average references after downsampling to 256 Hz. Finally, two 10 s continuous EEG data segments during and after painful stimulation were extracted, and every channel's data were transformed by the standardized z-score method. We transformed every voltage according to this expression z(t) = (x(t)–^−−^x)/σ and transformed the whole continuous data segment through 30 s data (10 s before stimulation, 10 s during stimulation and 10 s after stimulation).

### Quantitative EEG Analysis

Frequency domain analysis (power spectrum), nonlinear analysis (entropy) and brain network connectivity analysis (coherence and phase lag index) were used in subsequent analyses.

#### Power Spectrum Analysis

Processed time-series data were transformed into the frequency domain by a 1,024-point fast Fourier transform with Welch's method. Specifically, data were analyzed with a 512-point moving window with a 256-point overlap. Windowed data were extended to 1,024 points by zero-padding to calculate power spectrum, yielding an estimation of the power spectrum from 0.5 to 45 Hz (frequency resolution: 0.25 Hz). The power spectrum of these windows were averaged. The frequency bands were divided into delta (δ: 1–4 Hz), theta (θ: 5–7 Hz), alpha (α: 8–13 Hz), beta (β: 14–29 Hz) and gamma (γ: 30–45 Hz). The absolute power spectrum of a specific frequency band is the area under the power spectral density curve. The relative power spectrum of each frequency band is divided by the absolute power spectrum of the corresponding frequency band by the absolute power spectrum of the total frequency band (19). The relative power spectrum was calculated in this study.

#### Entropy Analysis

Sample entropy (SaEn): SaEn measures the degree of irregularity and predictability in a time series (20, 21). This method is derived from approximate entropy (ApEn), However, it can reduce the error of the ApEn calculation. The definition of SaEn is explained by combining the algorithm steps. Let the original data be *x*(1), *x*(2), ...*x*(*N*), a total of N points.

Step 1: Form a set of m-dimensional vectors in sequence of numbers as follows:
  $$\begin{array}{l} X_{i}^{m}=\left[x\left(i\right),x\left(i+1\right),...x\left(i+m-\;1\right)\right], \end{array}$$
  where 1 < *i* < *N* − *m*.
Step 2: Define $d\left[X_{i}^{m}-X_{j}^{m}\right]$ as the largest difference between *X*(*i*) and *X*(*j*) as follows:
  $$\begin{array}{l} d\left[X_{i}^{m}-X_{j}^{m}\right]=max^{0\leq k\leq m-1}\left(\left[x\left(i+1\right)-x\left(j+k\right)\right]\right). \end{array}$$
Step 3: Given a threshold *r*, count the number of $d\left[X_{i}^{m}-X_{j}^{m}\right]$ of each *i*-value less than the number of *r*. Then, calculate the ratio of this number to the total number of distances *N - m* + *1* as $B_{i}^{m}\left(r\right)$ as follows:
  $$\begin{array}{l} B_{i}^{m}\left(r\right)=\frac{\sum_{j=1,j\neq i}^{N-m}\theta \left(r-d\left[x_{i}^{m}-x_{j}^{m}\right]\right)}{N-m-1},1\leq j\leq N-m \end{array}$$
  where θ(*x*) is the Heaviside function.
Step 4: Take the average of $B_{i}^{m}\left(r\right)$ for all i; then, this value can be denoted as *B*^*m*^(*r*) as follows:
  $$\begin{array}{l} B^{m}\left(r\right)=\frac{\sum_{i=1}^{N-m}B_{i}^{m}\left(r\right)}{N-m} \end{array}$$
Step 5: Similar to steps 1~4, we define the function *A*^*m*^(*r*) as the total number of template matches under the embedding dimension of *m* + *1*.
Step 6: In theory, the SaEn of the sequence is the following:
  $$\begin{array}{l} SaEn\left(m,r\right)=-\text{ln}\frac{B^{m}\left(r\right)}{A^{m}\left(r\right)} \end{array}$$
  SaEn is obviously related to the value of *m, r*. Based on experience, we usually take *r* = *0.1*~*0.2 SD* (*SD* represents the standard deviation of the sequence *{x(i)}*). In this paper, SaEn has more reasonable statistical characteristics. Therefore, we set *m* = *3* and *r* = 0.2^*^SD.

Permutation entropy (PeEn): Similar to SaEn, PeEn is also used to estimate the regularity of time series signals and is based on the phase-space reconstruction method (22). In the PeEn algorithm, the data of the vector quantity $X_{i}^{m}$ are mapped to a sequence of a symbol, the probability density function is determined, and the value of PeEn is calculated. Compared to SaEn, PeEn has two advantages: first, it requires little time and works with small datasets; second, because it relies only on the relative value of data, the algorithm can reduce noise to a certain degree (23).

The definition of PeEn is explained by combining the algorithm steps. Let the original data be *x(1), x(2), : : :, x(N)*, a total of *N* points.

Step 1: Form a set of *m-*dimensional vectors in sequence of numbers as follows:
  $$\begin{array}{l} X_{i}^{m}=\left[x\left(i\right),x\left(i+1\right),...x\left(i+m-\;1\right)\right], \end{array}$$
  where *1* < *i* < *N-m*.
Step 2: Count the sort order of the sequence $X_{i}^{m}$, which has a total of *m*! for a vector quantity of *m* dimension. One of those arrangement patterns is π_*j*_(1 ≤ *j* ≤ *m*!), and its probability occurrence is defined as follows:
  $$\begin{array}{l} p\left(\pi_{j}\right)\frac{\sum_{i=1}^{N-m+1}\left\{X_{i}^{m\prime }s\;arrangement\;pattern\;is\;\pi_{j}\right\}}{m!\left(N-m+1\right)} \end{array}$$
Step 3: In theory, the ApEn of the sequence is the following:
  $$\begin{array}{l} PeEn\left(M\right)=-\overset{m!}{\underset{j=1}{\sum }}p\left(\pi_{j}\right)\log p\left(\pi_{j}\right) \end{array}$$
  PeEn is obviously related to the value of *m*. Considering the validity and complexity of the calculation, we usually take *m* = *3*~*10* based on experience (24). We set *m*=*3* in this paper. Thus, we can obtain a total number of *m!*=*6* in PeEn's arrangement pattern: π =*[012, 021, 102, 120, 201, 210]*.

#### Coherence

As a commonly used method, EEG coherence quantifies the linear correlation between two electrode locations' signals in the frequency domain by estimating the covariance of the EEG spectral activity (25). Mathematically, the coherence function *C*_*xy*_(*f*) at a frequency *f* for signals *x* and *y* is obtained by the normalization of the cross-spectral spectrum as follows:


$$\begin{array}{l} C_{xy}\left(f\right)=\frac{|Im\langle S_{xy}\left(f\right)\rangle {|}^{2}}{\sqrt{|\langle S_{xx}\left(f\right)\rangle {|}^{2*}|\langle S_{yy}\left(f\right)\rangle {|}^{2}}} \end{array}$$

where the notation 〈·〉 denotes the mean value over epochs of the time series. *S*_*xx*_(*f*) denotes the power spectral density estimate of signals x and *S*_*yy*_(*f*) denotes the power spectral density estimate of signals y, when *S*_*xy*_(*f*) represents the cross power spectral density estimate of signals *x* and *y, Im* is the operation of extracting the imaginary part of the complex number:


$$\begin{array}{l} S_{xy}\left(f\right)=F_{x}\left(f\right)\times F^{y*}\left(f\right) \end{array}$$

where *F*_*i*_(*f*), *i* ∈ {*x, y*} is the Fourier transform of signal *i, f* is the discrete frequency, and the symbol ^*^ denotes the complex conjugation.

The coherence value ranges from 0 to 1, where 0 indicates that the corresponding frequency components of both signals are linearly independent, and 1 indicates that the frequency components of the two signals give the maximum linear correlation. Furthermore, high EEG coherence indicates high cooperation and more information transmission between the underlying brain regions. After obtaining the function of the cross-spectral spectrum, we calculated the average coherence in the following frequency bands: delta (δ: 1–4 Hz), theta (θ: 4–8 Hz), alpha (α: 8–13 Hz), beta (β: 13–30 Hz) and gamma (γ: 30–45 Hz). The specific calculation equations are shown in the Supplementary Materials.

#### Phase Lag Index

Phase synchronization is an algorithm to estimate the dynamic synchronization index by calculating the synchronization of two signal phases in a frequency band (26). The calculation of phase synchronization is explained by the following steps:

Step 1: The continuous EEG data were filtered into 5 frequency bands: delta (δ: 1–4 Hz), theta (θ: 4–8 Hz), alpha (α: 8–13 Hz), beta (β: 13–30 Hz) and gamma (γ: 30–45 Hz).
Step 2: Filtered EEG data were transformed to complex analytical signals by using the Hilbert transform. A signal series *x* is transformed to an analytic signal by using the following:
  $$\begin{array}{l} Z_{x}\left(t\right)=x\left(t\right)+i\hat{x}\left(t\right)=A_{x}\left(t\right)e^{-iw_{x}\left(t\right)} \end{array}$$
  $\hat{x}\left(t\right)$ is the Hilbert transform of *x(t)*:
  $$\begin{array}{l} \hat{x}\left(t\right)=x{\left(t\right)}^{*}\frac{1}{\pi t}=\int_{-\infty }^{+\infty }x\left(\tau \right)\frac{1}{\pi \left(t-\tau \right)}d\tau \end{array}$$
  where ^*^ denotes convolution.
Step 3: We can obtain the instantaneous phase by using the analytic signal:
  $$\begin{array}{l} w_{x}\left(t\right)=\arctan\left(\frac{\hat{x}\left(t\right)}{x\left(t\right)}\right) \end{array}$$
  In order to obtain reliable estimates of phase synchronization that are invariant against the presence of common sources (volume conduction and/or active reference electrodes in the case of EEG), we introduced phase lag index (PLI). An index of the asymmetry of the phase difference distribution can be obtained from a time series of phase differences Δφ(*t*_*k*_), *k* = 1…*N*, in the following way:
  $$\begin{array}{l} PLI=|\langle sign\left[\phi \left(t_{k}\right)\right]\rangle | \end{array}$$
  The PLI ranges between 0 and 1. A PLA of zero indicates either no coupling or coupling with a phase difference centered around 0 mod π. A PLA of 1 indicates perfect phase locking at a value of ϕ different from 0 mod π. The stronger this nonzero phase locking is, the larger PLI will be (27).

## Statistical Analysis

Statistical analysis was performed using SPSS version 22.0 (IBM Corporation, Armonk, NY, USA) and MATLAB software. Categorical variables between the two groups were compared using chi-squared tests. A two-tailed *t*-test for normally distributed continuous variables was performed, and the Mann–Whitney U test was used in cases where the variable was not normally distributed. The permutation test was used for small sample data with an unknown population distribution and for hypothesis-testing problems that are difficult to analyze by conventional methods. By replacing the sample order, the statistical test quantity is recalculated to construct the distribution probability of the mean value, and then the *P*-value is calculated and inferred on this basis. *P* < 0.05 was considered statistically significant. Additionally, we calculated the false discovery rate (FDR) across the traditional statistics among the different parameters to reduce the number of false positives.

## Results

### Patient Baseline Data

**Figure 1:**
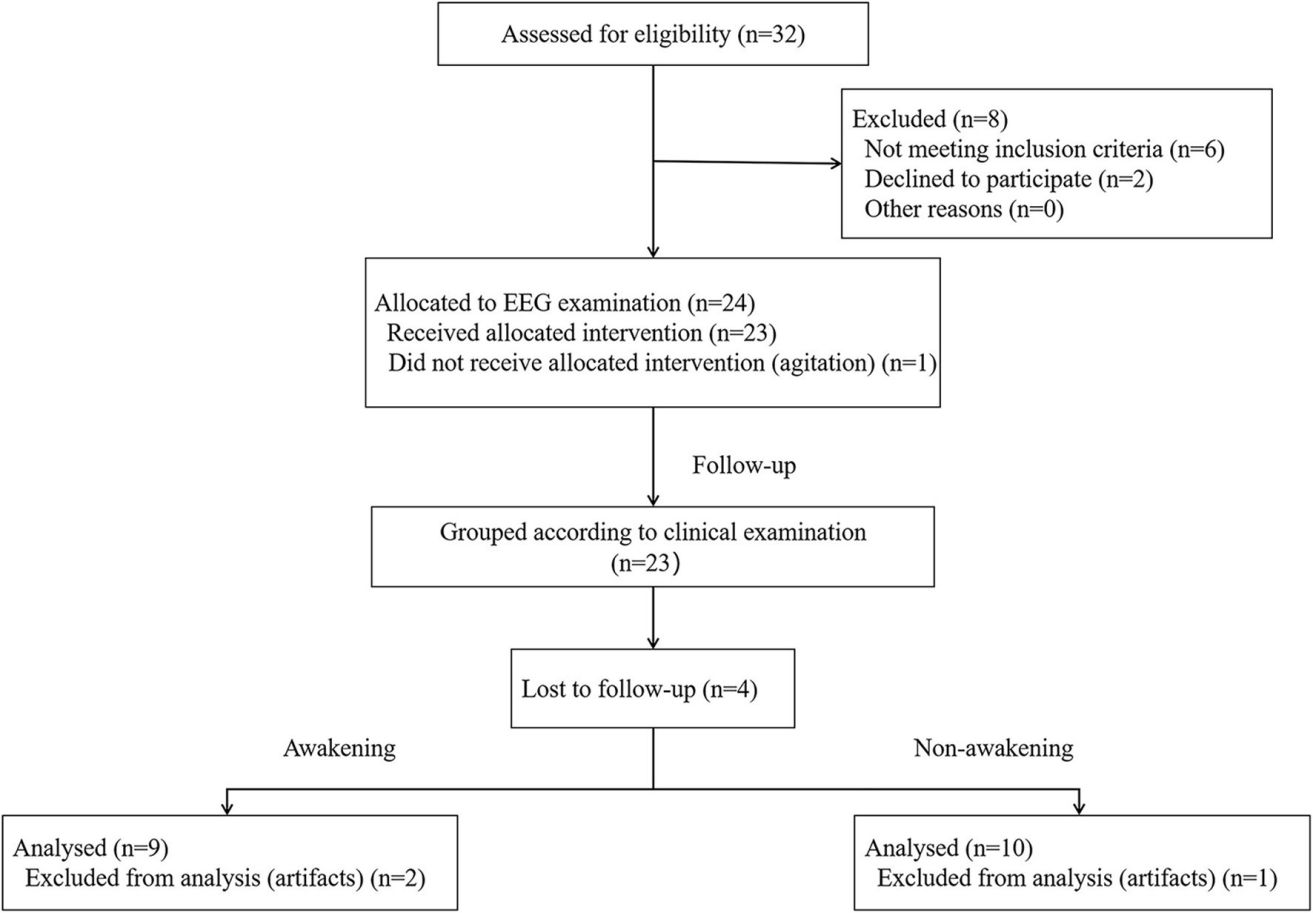
Flow diagram of the patient screening and exclusion criteria. EEG, electroencephalography.

From 2019 to 2020, a total of 16 comatose patients after CPR were included in the study (Figure 1). The time of ischemia and hypoxia was 3~20 (median: 5) min; EEG evaluation was 4~30 (median: 16) d from the onset time; GCS score was 3~8 (median: 6) points during EEG evaluation; 7 patients (43.8%) awakened within 3 months after coma, and 9 patients (56.3%) had not awakened by 3 months after coma. There was only significant difference in GCS score and CPC after 3 month between awakening and unawakening group (Table 2).

**Table 2:** Baseline data of patients.

ID, sex^*^, age	**Duration of arrest or resuscitation**	**Time from coma onset to EEG assessment (d)**	EEG classification^**^	**GCS score**	Outcome after 3 month^***^	**CPC after 3 month**
1, M, 68	4	14	S	7	A	2
2, M, 49	5	17	D	7	A	2
3, M, 83	10	13	D	6	A	2
4, M, 23	8	25	D	7	A	2
5, M, 53	10	16	S	4	A	3
6, F, 77	3	28	S	4	A	3
7, M, 59	5	19	D	7	A	3
8, M, 45	5	4	S	5	U	4
9, M, 68	4	6	D	3	U	4
10, M, 83	5	7	S	3	U	5
11, F, 34	4	30	D	5	U	4
12, M, 48	15	17	D	3	U	4
13, M, 62	4	29	S	5	U	4
14, F, 40	7	11	BS	3	U	4
15, M, 39	20	6	S	3	U	4
16, M, 85	4	22	S	5	U	4
Median, range	5, 3~20	16, 4~30		6, 3~8		4, 2~5
Z or χ^2^	−0.16	−0.95	1.415	−2.57		−3.60
*P*	0.87	0.34	0.49	0.01		0.00

* *M, male; F female*.

** * EEG, electroencephalography; S, suppression; D, delta/theta>50% of record (not theta coma); BS, burst-suppression*.

*** * A, awakening; U, unawakening; CPC, cerebral performance category*.

### Quantitative EEG Analysis

#### Spectral Power Analysis

**Figure 2:**
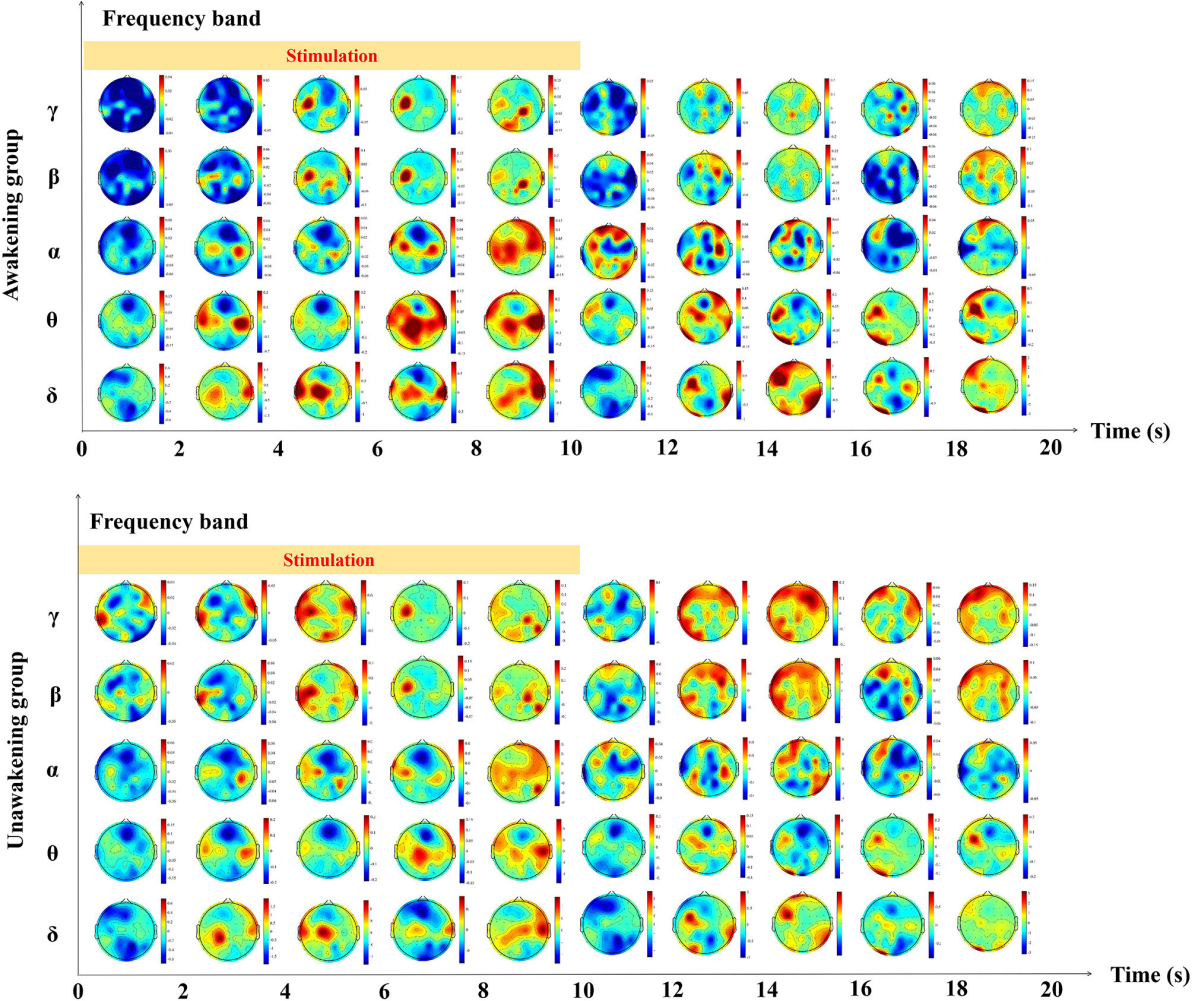
The dynamic changes of the relative power spectrum along the time.

**Figure 3:**
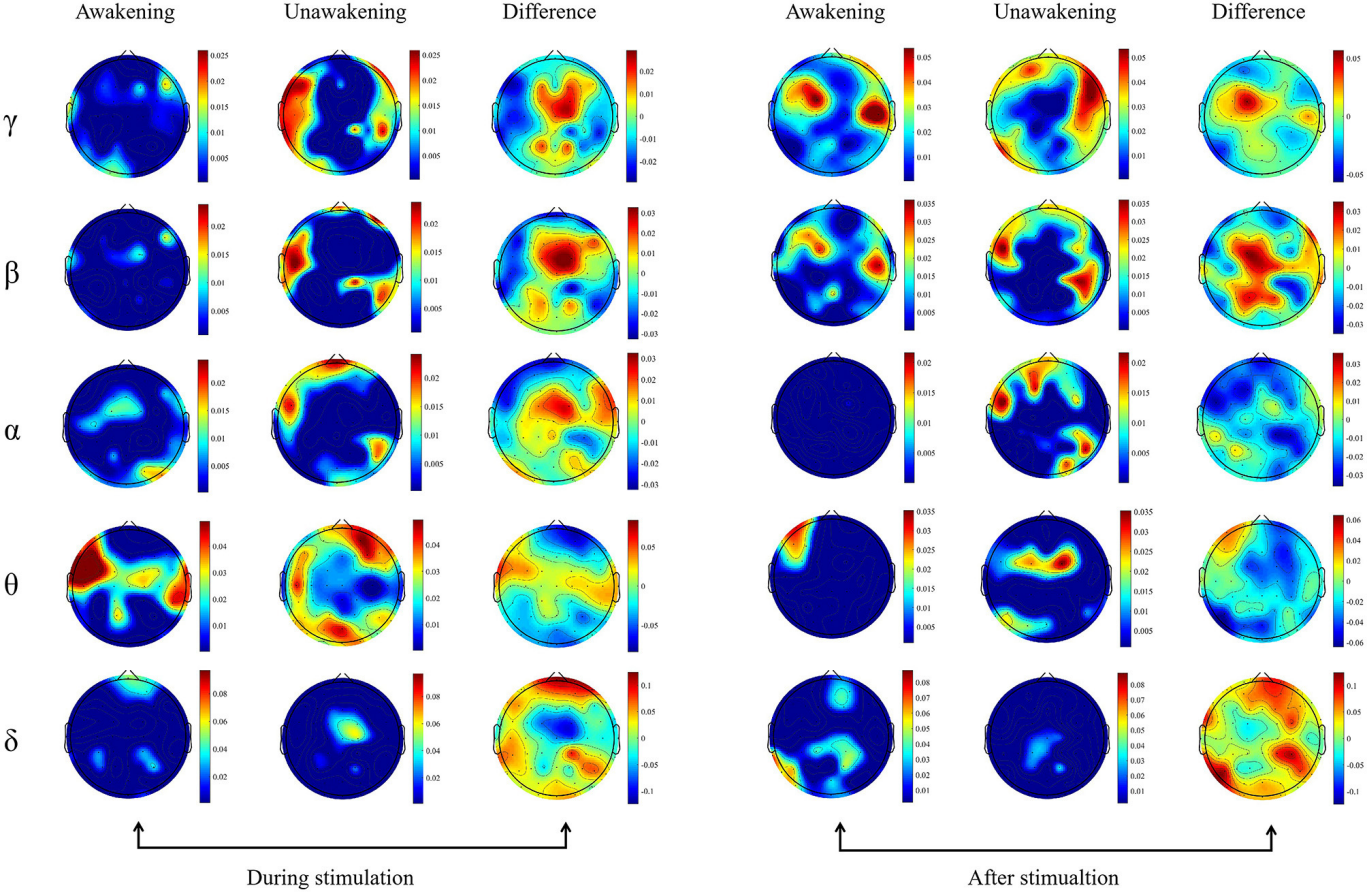
Topography based on relative spectral power. The spectral power between the awakening and unawakening patients were compared in different frequency bands during and after stimulation separately. The first column includes the results of comparisons before and during stimulation in the awakening group, the second column includes the results of comparisons before and during stimulation in the unawakening group, and the third column includes the difference between the awakening and unawakening groups. Similarly, the fourth, fifth and sixth columns are the results of comparisons after stimulation and between stimulation in the awakening group and unawakening group, respectively (fourth and fifth columns) and their difference (sixth column). The color bar represents the actual value of the relative power spectrum.

We calculated the dynamic changes along the time of power spectrum as showed in Figure 2. In the awakening group, γ and β frequency band relative spectral power in the frontal and parietal lobes, α frequency band relative spectral power in the central lobe, θ and δ frequency band relative spectral power in the temporal and occipital lobes increased gradually during the process of pain stimulation. In the first 2 s after pain stimulation, there were sudden decreases in all frequency bands relative spectral power in nearly the whole brain and gradually recovered later. In the unawakening group, the trend of dynamic changes seemed to be similar to the awakening group, therefore we further compared the differences between the two group. In the process of pain stimulation, compared with the unawakening group, the power spectrum changes in the awakening group compared with the unawakening group mainly manifested in the central brain cortex (parietal lobe) as an increase in γ, β and α frequency band relative spectral power; in the bilateral temporal lobes, θ frequency band relative spectral power increased, and in the bilateral frontal and occipital lobes, δ frequency band relative spectral power increased. Ten seconds after pain stimulation, the awakening group had higher γ and β spectral power in the central brain region (parietal lobe) and higher δ frequency band spectral power in nearly the whole brain (with the exception of the central region). However, there were no significant differences in the above relative power spectrum changes (Figure 3).

#### Entropy

**Figure 4:**
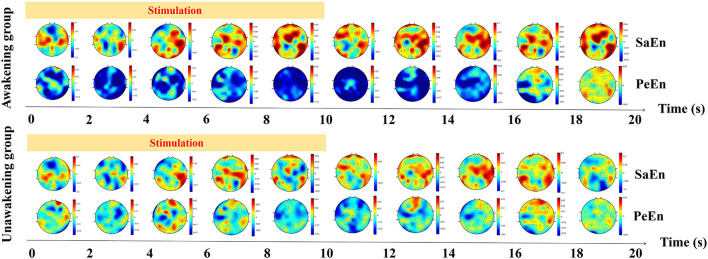
The dynamic changes of entropy along the time. SaEn, sample entropy; PeEn, permutation entropy.

**Figure 5:**
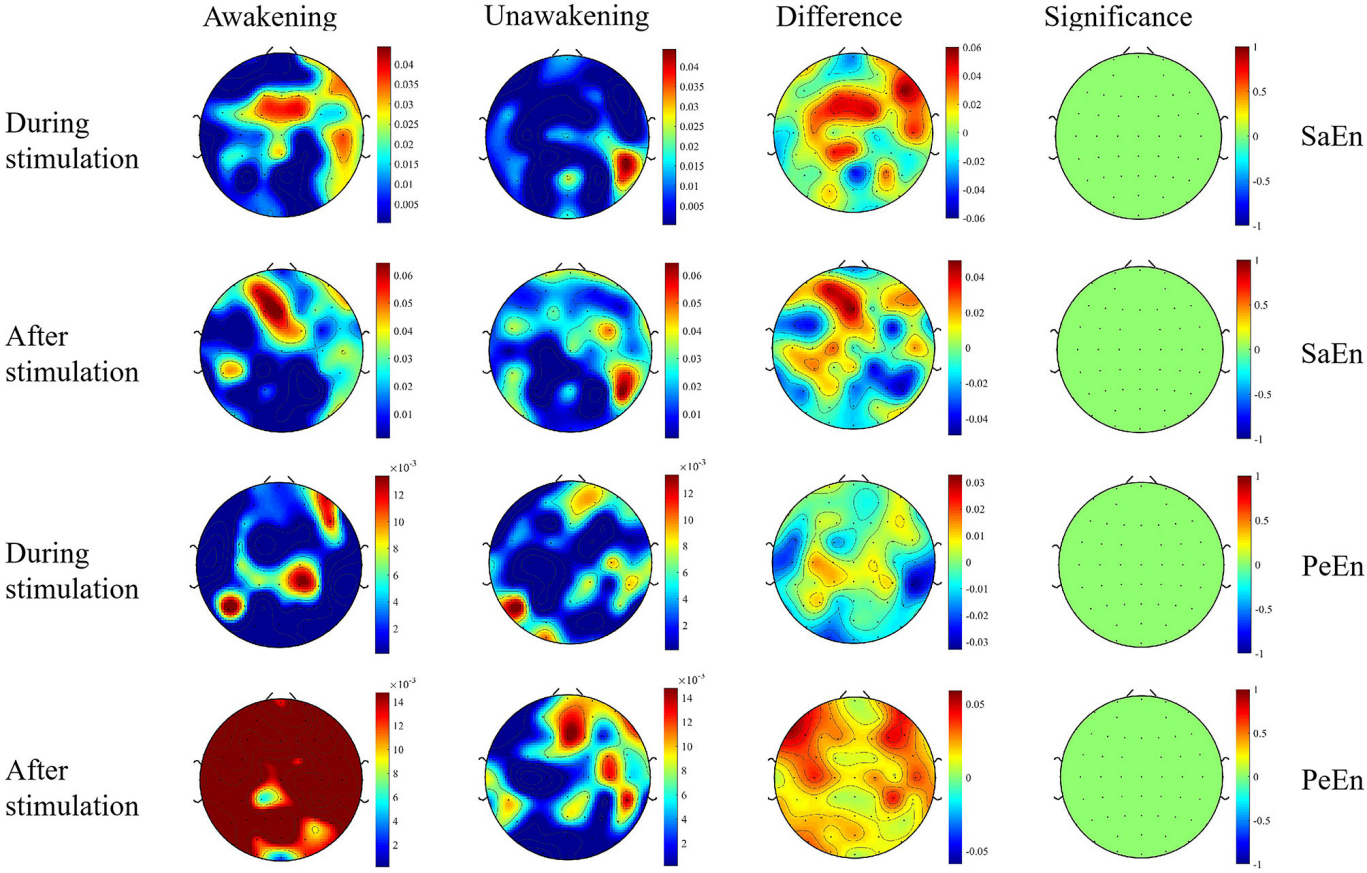
Topography based on SaEn and PeEn. Significance is the statistical *P*-value; the greater the absolute value of *P*, the greater the difference. The first column and second column are the results of comparisons before and during (or after) stimulation in the awakening group and unawakening group, respectively. The third column is a comparison between the awakening and unawakening groups. The color bars in the first three columns represent the actual values of the entropy; the color bar in the fourth column represents the statistical value t. SaEn, sample entropy; PeEn, permutation entropy.

The dynamic changes of entropy along the time was showed in Figure 4. In the awakening group, SaEn increased gradually in nearly the whole brain in the whole stimulation process and 10 s after stimulation while the PeEn didn't begin to increase until 6 s after stimualtion. In the unawakening group, SaEn and PeEn in the whole brain were also increasing gradually, but the changes were not obvious. We compared the differences between the two groups and the results showed in Figure 5. SaEn: The changes in SaEn in the awakening patients, compared with the unawakening patients, during stimulation were mainly an increase in the central brain area and decreases in the bilateral temporal and occipital lobes. Within 10 s of pain stimulation, the SaEn of the whole brain increased. Additionally, these changes were not significantly different. PeEn: The changes in PeEn in the awakening group, compared with the unawakening patients, during pain stimulation were mainly increases in the frontal lobe and parietal lobe, and there was a significant difference in the increase in the frontal lobe. The PeEn in the awakening patients remained increased in the frontal-parietal lobes within 10 s after pain stimulation, However, the changes were not significantly different.

#### Connectivity

##### Coherence

During the period of pain stimulation, the awakening group had higher γ and β coherence across the whole brain than the unawakening group, and the increase in β coherence in frontal-parietal and frontal-central lobes were more obvious. α and δ coherence in the whole brain, θ coherence in the parietal-occipital and parietal-temperal lobes were significantly decreased. After pain stimulation, γ and β coher ence in nearly the whole-brain area remained increased, the increase of β coherence was stronger and wider, and θ and δ coherence in the whole brain began to increase.

##### Phase Lag Index (PLI)

**Figure 6:**
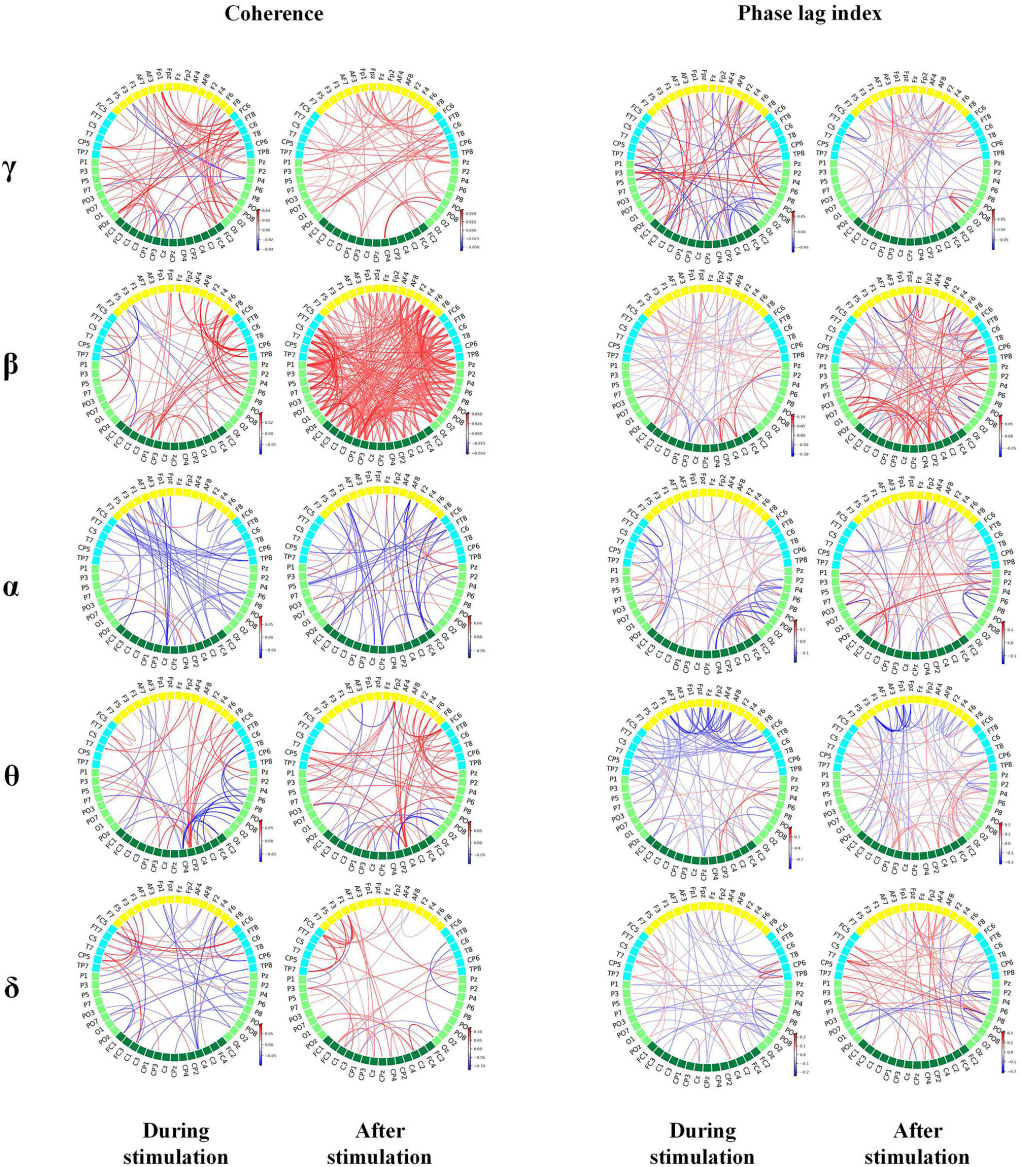
Brain connectivity map based on coherence and phase synchronization. The red line indicates stronger connections, while the blue line indicates weaker connections in awakening patients than in unawakening patients during and after stimulation. The color bar is the connectivity difference. Statistically significant connectivity differences are plotted in the figure.

During the period of pain stimulation, the awakening group had higher γ and β PLI across the whole brain than the unawakening group. α PLI in the central-parietal lobes, θ PLI in the frontal-central lobes and δ PLI in nearly the whole brain was mainly decreased. Within 10 s after stimulation, the increase of γ PLI was less, and whole-brain β PLI remained increased and stronger. α, θ and δ PLI began to increase in the whole brain when compared with unawakening group (Figure 6).

## Discussion

Leading neuroscientific theories posit a central role for the functional integration of cortical areas in conscious states. Therefore, we used EEG to continuously explore cortical functional integration. On the basis of previous studies, we used previously confirmed parameters related to consciousness (power spectrum and entropy) and connectivity (coherence and phase synchronization) to provide a further quantitative analysis of the reactivity characteristics of HIE patients after pain stimulation (13). Compared with the resting state, pain stimulation affects oscillations in the neural network. Through thalamocortical and corticocortical feedback, these oscillations can reflect the brain's ability to receive and process sensory information (28, 29).

Quantitative analysis (computer-based) can assist EEG analysts, increase the speed and accuracy of EEG interpretation, and find EEG features that cannot be easily recognized by visual analysis. Most quantitative analysis methods use “feature programming”; that is, accurately designed algorithms are used to detect or quantify predefined features of EEG signals, such as amplitude, frequency, discharge and linear or non-linear interactions between channels (30, 31). Our quantitative analysis found an initial increase in the spectral power of fast oscillations (α, β, and γ) in the parietal lobe during pain stimulation in patients who awakened compared with those who did not. Following the stimulation, the spectral power of the slow oscillations gradually increased, replacing the fast oscillation EEG. Similarly, an initial increase in entropy appeared in the frontal and parietal lobes and then spread to the whole brain. The increase in fast-frequency band energy after pain stimulation in the awakening group indicated that some cortical functions were retained. In the clinical and preclinical environment, increases in slow frequency spectral power are considered to be features of unconsciousness. The appearance of low-amplitude fast EEG, accompanied by the disappearance of slow oscillations, is related to awakening (32–34). Patients who awakened had higher entropy after pain stimulation, indicating that a greater likelihood of awakening was associated with more complex EEG signals and richer information content and information flow (22). Previous studies mostly focused on patients with chronic disorders of consciousness, and there have been few studies on acute coma, but our results were similar to those of chronic disorders of consciousness; that is, increases in fast oscillation spectral power and increases in spatiotemporal complexity were related to awakening (15, 35–37).

This study also found that after pain stimulation, changes in the power spectrum and entropy in the patients who later awakened first appeared in the frontal-parietal lobe. On the one hand, the primary somatosensory afferent center is located in the anterior parietal lobe in the central posterior gyrus. On the other hand, the frontal-parietal lobe may indeed play an important role in the formation of consciousness. The prefrontal cortex, parietal cortex, basal forebrain and anterior cingulate cortex play important roles in the recovery of impaired consciousness, which has been indicated in many studies based on positron emission tomography (PET), EEG and functional MRI (fMRI). In 2000, Laureys et al. observed that functional connectivity of the prefrontal lobe, anterior cingulate gyrus and thalamic nucleus in patients with vegetative state was lower than that in healthy controls using H2^15^O PET. With recovery of consciousness, functional connectivity of these areas gradually returned to normal (38). Several studies on disorders of consciousness based on EEG and fMRI found that increases in frontoparietal functional connectivity were positively correlated with improvements in coma recovery scale revised (CRS-R) scores (39–41). In 2020, Pal et al. found that delivery of carbachol to the prefrontal cortex induced wakefulness in mice subjected to continuous administration of the general anesthetic sevoflurane (42). In 2012, Leon et al. found that the disappearance or reduction in brain activity in the anterior brain (frontal-parietal lobes) would terminate or limit the processing of consciousness and believed that the anterior brain (frontal-parietal lobes) should play a more important role in the generation of consciousness, while the activity in the posterior head (occipital lobe) might reflect the rigid cortical reflex related to the content of consciousness (43). In 2021, Mashour et al. found that the frontal-parietal cortex, which is responsible for executive function, returned first in the recovery of consciousness in healthy individuals who received general anesthesia (44). Therefore, we believe that the degree of retention of frontal-parietal lobe function after brain injury may affect predictions of awakening in comatose patients.

Our study found that the awakening group showed increases in fast oscillation (γ and β) connectivity (coherence and phase synchronization) in nearly the whole brain and decreases in slow oscillation (θ and δ) connectivity in some brain regions. Regarding corticocortical connections, the higher the coherence and phase synchronization of EEG signals was, the stronger the potential neuronal coordination, and the better the retention of brain function (45). HIE after CPR causes whole-brain injury, which limits incoming afferent nociceptive sensory impulses to the cerebral cortex, and the ability of cortical neurons to synchronize or desynchronize is also restricted. Therefore, the decrease in coherence and synchronization after stimulation indicates that the integrity of the arousal system is seriously damaged and the possibility of arousal is significantly reduced.

Most studies that examined the predictive value and the reliability clinical outcome predictions in this context had the limitation of not blinding the neurologists to the EEG findings and not blinding the EEG analysts to the outcomes. To avoid information bias in the design, data collection and analysis stages, we used a blind method in the design, which had the major advantage of excluding “self-fulfilling prophecies”. There were still some limitations in this study. (1) The sample size was small, and the representativeness was poor. (2) Encephaledema was an important confounding factor. Within 1 month of onset, encephaledema has a great impact on the evaluation. Even with standard medical treatment, it was difficult to ensure that the degree of brain edema was the same in every patient. Thus it was hard to imagine that the recovery of cortical cognitive function in such patients was not partly due to the remission of encephaledema. (3) Pain stimulation by pressing the nail bed could not be quantified, and differences in stimulation intensity and duration may have introduced bias. Because we used only one stimulation per EEG, we could not ensure reproducibility, which is critical for distinguishing reactivity from EEG background fluctuations. These shortcomings are the main reasons why EEG reactivity to stimulation is less reliable than other clinical, electrophysiological, and neuroimaging predictors studied to date, as shown in many previous studies and outlined in the most recent systematic review on this topic (46). Hence, it is highly unlikely that the applied EEG analyses will compensate for these persistent shortcomings when the EEG reactivity characteristics are compared with other well-studied predictors in larger cohorts. Thus, quantifiable stimulation, such as electrical square-wave pulses or thermal stimuli, could be used as a tool for reactivity in future research to avoid such limitations. (4) The stimulation time of pressing was relatively short as power spectrum and entropy analysis need stable EEG state, that is, it needs a relatively long sequence. Too short data series would decrease its accuracy. (5) We recorded the bilateral cortical activation by pressing on either side of the nail bed. After we have accumulated a larger sample size, we will alternate the sides of the stimulation (left or right) to determine whether the results are different in future studies. (6) Electromyogram (EMG) artifact is another significant confounding factor. We removed the channel seriously affected by EMG, and then use the data of peripheral channels to fit a new data to replace the channel. However, EMG artifact contaminated nearly all EEG channels and there is a high spatiotemporal overlap between EMG artifacts and EEG signal. Existing methods to remove EMG artifacts include independent component analysis (ICA) and other high-order statistical methods. However, these methods can not effectively remove most of the EMG artifacts (47, 48). (7) The time between cardiac arrest and EEG measurements was not standardized (due to the small sample size), which was another important confounding factor since different time frames could produce different results. We will supplement our work with analyses in different time frames after a sufficient number of patients are admitted in our future research. (8) The spectrum of EEG malignant categories (suppression, burst-suppression, α and θ coma and generalized periodic complexes combined) is greatly variable, it is difficult to quantify different EEG features of malignant categories. Therefore, power spectrum had great shortcomings in evaluating disorders of consciousness. (9) EEG reflects only the functional activities of the cerebral cortex and not the functions of the subcortical cortex and brainstem, although wakefulness requires that the functions of the ascending reticular activation system of the cerebral hemisphere and brainstem are simultaneously intact. EEG has high temporal resolution but low spatial resolution. fMRI could compensate for the low spatial resolution of EEG. If fMRI could be added to future research, it could make predictions of awakening after coma more accurate. (10) The metrics studied in our research cannot yet be routinely used in daily medical treatments due to the time-consuming and complex calculations. (11) Additionally, we only found some change rules rather than a cut-off value or marker, and we did not compare the prediction efficiency of the parameters with other well-studied predictors (such as SSEPs, EEG background activity changes/patterns, and MRIs) (46).

## Conclusions

Our study found that if pain stimulation in comatose patients after CPR stimulates higher fast oscillation spectral power, higher entropy and stronger whole-brain connectivity and if the function of frontal-parietal lobe is better preserved, then these patients are more likely to awaken.

## Data Availability Statement

The data that support the findings of this study are available on request from corresponding author.

## Ethics Statement

The studies involving human participants were reviewed and approved by the Ethics Committee of Xuanwu Hospital, Capital Medical University, Beijing. Written informed consent for participation was not required for this study in accordance with the national legislation and the institutional requirements.

## Author Contributions

HH, YS, and GL were responsible for the conception and design of the study. Data acquisition was performed by HH and MJ. XL and ZN were responsible for the analysis and interpretation of data. YS, HH, and ZN were involved in drafting the article or revising it critically for important intellectual content. All authors approved the final version of this manuscript for publication. All authors contributed to the article and approved the submitted version.

## Conflict of Interest

The authors declare that the research was conducted in the absence of any commercial or financial relationships that could be construed as a potential conflict of interest.

## Publisher's Note

All claims expressed in this article are solely those of the authors and do not necessarily represent those of their affiliated organizations, or those of the publisher, the editors and the reviewers. Any product that may be evaluated in this article, or claim that may be made by its manufacturer, is not guaranteed or endorsed by the publisher.
